# Moving Toward Remote, Parent-Reported Measurements in Pediatric Anthropometrics for Research and Practice

**DOI:** 10.3389/fped.2022.838815

**Published:** 2022-03-08

**Authors:** Eliot N. Haddad, Tsoline Kojaoghlanian, Sarah S. Comstock

**Affiliations:** ^1^Department of Food Science and Human Nutrition, Michigan State University, East Lansing, MI, United States; ^2^Department of Pediatrics, Maimonides Children's Hospital, Brooklyn, NY, United States

**Keywords:** pediatrics, anthropometry, remote measurement, telemedicine, parental report

## Introduction

Anthropometry is the study of human anatomical measurements and can provide insight into growth, energy/nutritional intake, physical fitness, and clinical care (1). Common anthropometric measurements include body/head circumferences, height, weight, and skinfold thickness. Such anthropometric measurements underlie further proxies for health/growth such as body mass index (BMI).

The accurate attainment of body measurements and proportions is especially relevant to the field of pediatrics, where growth occurs rapidly and must be quantified in reliable and valid fashions. Anthropometry in pediatrics aids in the diagnosis of such conditions as stunting, wasting, congenital/acquired hormonal disturbances, brain development, and malnutrition (1). For example, the mid-upper arm circumference allows for insight into the nutritional status of a child or a pregnant woman. Similarly, length/height-for-age, weight-for-age, weight-for-length/height, and BMI-for-age are all useful as growth indicators in pediatric populations. These metrics are best reported as z-scores (number of standard deviations away from a reference median/mean) due to their ease of interpretation and ability to be statistically summarized (mean, median, quartiles) (2). In research, these data can be applied in epidemiological models to identify potential associations that can inform public policy to address population-level nutritional or lifestyle deficits. Clinically, anthropometric measurements directly inform diagnosis and assessment. Herein, we discuss how to approach remote pediatric anthropometry for research and healthcare during a pandemic, where physical engagement is limited.

## The Rise of Remote Anthropometry

Anthropometric data can be collected with the use of different tools and methods depending on the body site in question. For height and weight, a tape measure and scale are normally sufficient to obtain workable measurements, though a stadiometer is the ideal instrument for measuring height (3). In pediatric populations, especially those under 2 years, it is the length, rather than height, that is measured. Length measures in infants are often challenging to accurately obtain since the child must lie in a supine position for the duration of the measurement, which usually necessitates the help of an additional person. Additionally, skin folds and body circumferences are often more difficult to obtain due to their reliance on more intricate tools and nuanced procedures. For example, it may be unclear at which locations to measure circumferences and how to correctly use a caliper for an untrained individual. Due to this, a specialist is usually required for the attainment of anthropometric data other than height and weight.

Though in-house clinical measurements by an anthropometric specialist are regarded as the gold standard of anthropometry (3), remote methods for height/weight measurement are becoming increasingly popular due to their recognized benefits of cost-efficiency on the part of researchers and participants alike, especially in the wake of the COVID-19 pandemic (4). For adults, several studies have validated self-reported height and weight measurements (5). However, in pediatric populations, parental self-reporting of anthropometrics is more prone to error due to the inherent difficulties of conducting the necessary procedures on a child or infant (2).

A retrospective study conducted during the current COVID-19 pandemic in a single pediatric endocrinology center demonstrated that remote measurements among a cohort ranging from 3 to 18 years could be clinically useful due to their overall good concordance with in-person measurements (6). Interestingly, remote, parent-provided measurements of overweight and obese children were deflated for height. For all participants, weight tended to be deflated when reported by parents. This phenomenon has been demonstrated in other studies as well and seems to increase with child age (7–9), perhaps due to social desirability bias, lack of parent knowledge regarding proper measurement procedures, or imprecise scales. Nonetheless, with proper guidance, education, and adherence to best practice instructions, remote collection of pediatric anthropometric data is bound to become not just more commonplace, but also relied upon in clinical decision-making, especially in light of infectious disease pandemics that have forced ongoing health checks to transition to remote formats (4).

## Lack of Validation in Infant Populations

Remote pediatric anthropometrics are typically reported by a parent/guardian of the infant or child and may be obtained in many ways. The first method of remote anthropometric measurement entails guiding a parent/caregiver in the process of measuring their child's body. This may occur live via videoconference and/or through an instructional pamphlet, available in multiple languages, that provides adequate detail for the parent to obtain measurements effectively and accurately. Such guidance has been shown to significantly improve parental measurements and increase the accuracy of pediatric BMI categorization based on remote measurements (10). This guided method of measurement has relevance for both research and clinical practice, though this assumes that the parent will have access to the necessary tools to collect such measurements. Indeed, no degree of guidance can overcome an absence of equipment, which is yet another consideration to be made when utilizing remote anthropometry. A parent may report pediatric anthropometrics based on an estimate or an unguided home measurement, but this is prone to much error and is unlikely to provide constructive information for clinical use (11).

In research studies, remote anthropometric data can be collected by simply taking measures from the child's medical records. Parental report based on recall from the last clinical visit is also a viable option. However, this may introduce recall bias or cause the reported measurements to be outdated depending on when the last visit occurred. This is especially true for infants, who grow swiftly in the first year of life. Though not yet widespread, there are also several technologies being developed that can allow for the collection of anthropometrics by way of three-dimensional scanning in mobile clinical/research settings (12). These novel methods synergize with telematics by allowing measurement data to be directly logged and transferred to physicians or researchers.

For infants especially, there are physical challenges involved in collecting measurements, even in a professional setting (2). Weight measurements for children under 2 years of age are typically collected using baby scales that provide a platform on which the infant can be set down. Occasionally, for infants who are particularly squirmy or agitated during the weighing procedure, a parent is weighed while holding the infant, then the parental weight is subtracted from that measure to obtain the infant weight. For length, infants are classically measured while recumbent on a specialized board that has a fixed headpiece and mobile footpiece. Through all this, infants often cry and kick, sometimes necessitating two persons to obtain accurate data. Hence, obtaining remote measurements for infants is particularly challenging, especially considering the specialized equipment that is usually necessary. Even in clinical settings, it is not uncommon for a documented measurement to be corrected during the same visit after the care provider plots the value in the infant's growth chart, realizes that the growth curve has an unexpected shape, and decides to take a second measurement. Older children can be measured for height and weight relatively easier than infants, since household scales and measuring tapes will ordinarily be sufficient for accurate measurements, though there is the possibility of systematic or random errors arising from the equipment or measuring techniques (6, 13). If these household tools are applied to infants for anthropometric measurements, the errors may further compound. Moreover, if a child or infant has a disease that impacts their motor function or attention span, measurement may be greatly hindered, especially for those parents who are not trained in the proper measurement procedures. Parents are deeply invested in their infant's growth, often citing actual numbers for weight and length to friends and family as a proxy for their child's health (14, 15). Thus, it is expected that parental interest and participation in learning and utilizing remote methods will be high. However, this may concurrently lead to social desirability confounding in the reported measures if faster growth is perceived as favorable by the parents and their social circles (16). Hence, validation of parent-reported values of infant anthropometric measurements is imperative considering the likelihood that remote methods will continue to proliferate in pediatric research as well as practice and given their potential to shed light on growth in geographically dispersed and medically underserved populations.

Because of these described challenges posed by pediatric anthropometrics, parental reports have not always been concordant with measurements obtained by a specialist (4). However, in envisioned clinical practice in the future and for large research studies, parental report is often the most logistically feasible method. Additionally, parent-reported measurements have value in their ability to promote participation and inclusion because participants need not live near clinical centers to participate. Remote anthropometry also enables the process of data collection for geographically dispersed and medically underserved populations who may not have access to or resources for in-person check-ups or care. Additionally, during infectious disease outbreaks, including the current pandemic, remote anthropometric measures can help reduce potential disease exposure by reducing time in clinic and facilitating remote health tracking through telemedicine (17, 18). For socially vulnerable families and for parents of children experiencing disease and disability, the benefits of remote anthropometry have the potential to outweigh the initially perceived and actual challenges, considering the likelihood for saving them time, money, and stress. These challenges include limited economic resources, lack of insurance, cultural barriers (language, mannerisms, social expectations), and health illiteracy, but can be partially overcome with virtual care strategies, according to the American Hospital Association (19).

## Discussion

**Figure 1:**
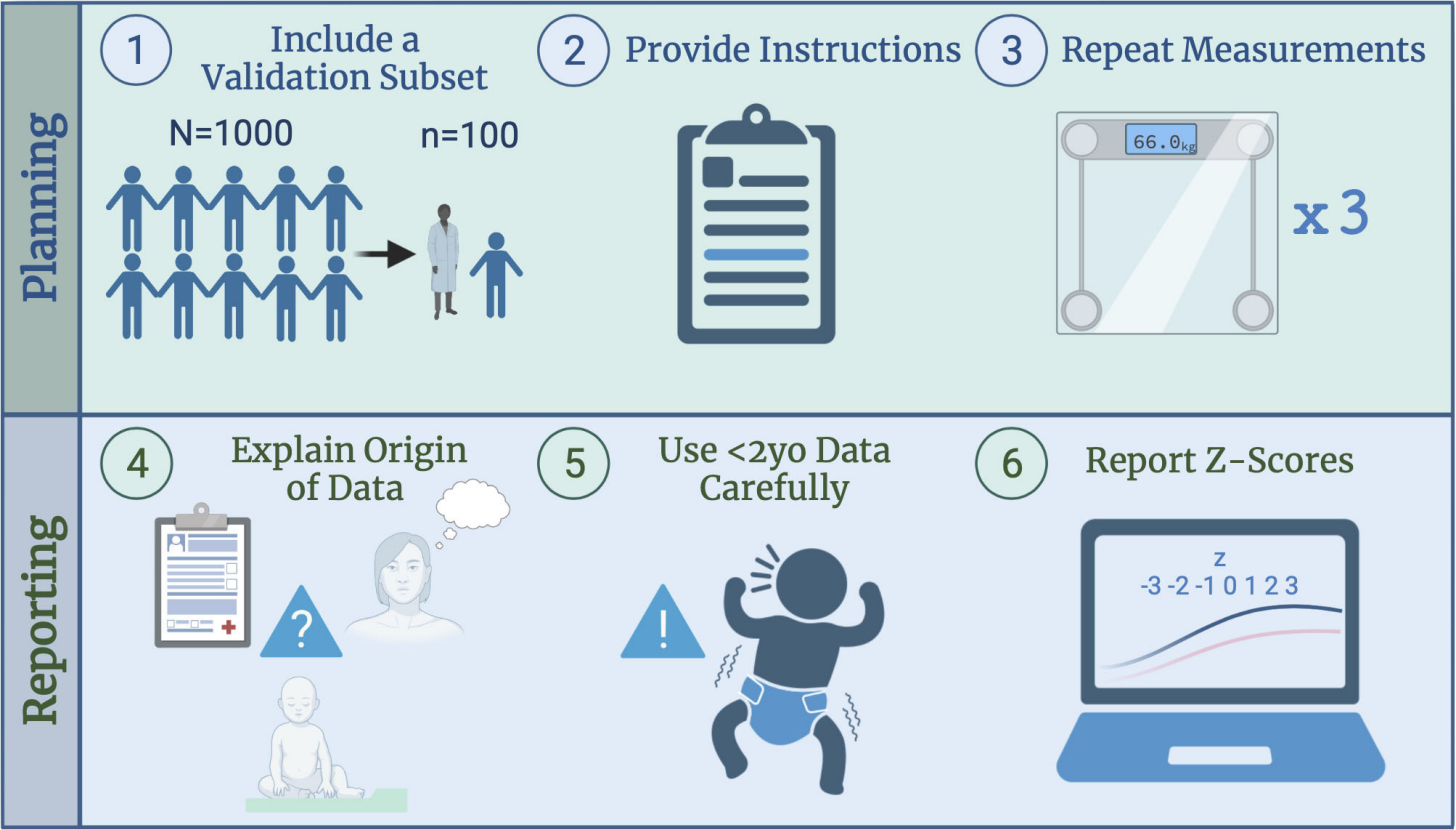
Factors to consider when designing a study including remote, parent-reported infant length and weight measurements.

Remote, parent-reported measurements of pediatric anthropometrics have been validated in children over 2 years old. With varying results, most show some degree of concordance between parent-reported and clinical metrics, especially in older age groups (20). However, there are several considerations both scientists and clinicians can make when conducting research or utilizing results that have incorporated parent-reported data (Figure 1).

### Planning Research

1. Consider validating parent-reported measurements within a study by taking a representative sample of participants and comparing with specialist-obtained values. This can be especially useful if the population is geographically/ethnically/habitually unique, since existing validations are often based on populations that are not representatively diverse (20).
2. Provide parents/caregivers with a set of instructions regarding the desired method of anthropometric measurement. Instruct collection of measurements to the nearest tenth to limit heaping of parent-reported values at multiples of 0.5, which limits accuracy (2). Consider providing parents of infants with instructions to make or obtain equipment necessary for accurate measurements (21), such as length boards or baby scales.
3. Have participants repeat measurements at each timepoint so that the average can be used for downstream analysis or clinical input (4). Data collected in triplicate will attenuate random error.

### Reporting Research

4. Be clear in addressing the method by which child/infant caregivers derived the reported measurements (11)—i.e., Was it recall from a previous primary care visit? Was it an estimation? Was it measured by the parent themself? What type of instructions were provided?
5. Exercise caution when utilizing parent-reported measures of infant (<2 years old) length and weight (22). The lack of validation studies in the existing literature is a gap that should be filled. Use remote measures as a back-up option if a patient population is inaccessible due to geography/circumstances, or quick nutritional/growth screening for rural populations is required (16).
6. For pediatric populations, report anthropometrics as a z-score, since this method accounts for age and sex of the participants (2). Standalone height and weight values have limited analytical and interpretative value due to the highly variable growth rates and body sizes of infants and children.

## Conclusion

Anthropometry is key to understanding the health of a population, and regarding children/infants, remote methods can be especially important for streamlining research, offsetting costs/burden, and improving accessibility to promote inclusion of diverse populations. As the field moves toward more widespread use of remote anthropometry, validation of these measurements is necessary to ensure they retain clinical and practical translatability.

## Author Contributions

EH contributed to conceptualization, investigation, visualization, and writing—original draft. TK contributed to validation and writing—review and editing. SC contributed to conceptualization, project administration, supervision, validation, and writing—review and editing. All authors have read and approved the final manuscript.

## Conflict of Interest

The authors declare that the research was conducted in the absence of any commercial or financial relationships that could be construed as a potential conflict of interest.

## Publisher's Note

All claims expressed in this article are solely those of the authors and do not necessarily represent those of their affiliated organizations, or those of the publisher, the editors and the reviewers. Any product that may be evaluated in this article, or claim that may be made by its manufacturer, is not guaranteed or endorsed by the publisher.
